# Spontaneous Remission in Hashimoto’s Encephalopathy: A Report of a Rare Case

**DOI:** 10.7759/cureus.100975

**Published:** 2026-01-07

**Authors:** Mohamed Hersi, Abdulahi Werar, Mohamed Guled, Naima Ragge, Abdirahman Mohamed, Mohamed Issa, Abdirashid Ali, Kedir Hamid

**Affiliations:** 1 General Internal Medicine, Luton and Dunstable University Hospital, Luton, GBR; 2 Internal Medicine, Barking, Havering and Redbridge University Hospitals NHS Trust, London, GBR

**Keywords:** antithyroid antibodies, autoimmune encephalopathy, corticosteroids, hashimoto’s encephalopathy, seizures, spontaneous remission

## Abstract

Hashimoto’s encephalopathy is a condition usually diagnosed by exclusion. Although its exact aetiology remains unknown, extensive case reports and studies suggest that autoimmunity contributes to its development with the presence of antithyroid antibodies. While there is no pathognomonic sign of Hashimoto’s encephalopathy, it manifests with encephalopathic symptoms such as confusion, delirium, and even seizures.

This case report discusses a 35-year-old woman with a notable history of hypothyroidism who presented with seizures preceded by a day of episodes of confusion and unresponsiveness. Upon admission, she was transferred to our intensive care unit. Extensive investigations revealed elevated thyroid peroxidase antibodies and an electroencephalogram (EEG) showing wave patterns characteristic of an encephalitic process. The patient met the diagnostic criteria for Hashimoto’s encephalopathy after excluding other potential causes. The patient's symptoms were well controlled with levetiracetam and phenytoin, and her thyroid dysfunction was treated with levothyroxine, resulting in subsequent improvement.

The primary treatment for Hashimoto’s encephalopathy is corticosteroids. Literature highlights corticosteroids as the main treatment for Hashimoto’s encephalopathy, although alternative options such as intravenous immunoglobulin (IVIG) and plasma exchange have also been used. Nonetheless, despite its rarity, there have been reported instances of spontaneous remission. Our case similarly illustrates this possibility, as we discuss how a young female patient who meets the criteria for Hashimoto’s encephalopathy experienced complete recovery through spontaneous remission. Clinicians should therefore be aware of the potential for spontaneous remission when dealing with Hashimoto’s encephalopathy.

## Introduction

Hashimoto’s encephalopathy is a disorder with autoimmune components, characterised by the presence of elevated antithyroid antibodies and its responsiveness to steroid therapy. Patients diagnosed with Hashimoto’s encephalopathy may present with a variety of symptoms, including seizures, confusion, and behavioural changes [1]. The diagnostic criteria for Hashimoto’s encephalopathy, as suggested by Mattozzi et al. [2], include the presentation of an altered mental state and/or seizure-like activity, coupled with the presence of antithyroid antibodies. The criteria will be met in the absence of underlying physical causes such as a stroke, tumour, or central nervous system infection. Furthermore, Mattozzi et al. [2] have indicated that individuals with Hashimoto’s encephalopathy are expected to be in a euthyroid or hypothyroid state and will demonstrate normal brain MRI results or nonspecific changes. However, a few patients in their study showed “increased T2 signalling and hyperintense lesions” on their brain MRI scans [2].

## Case presentation

We examine the unique case of a 35-year-old female who was found to be unwell, exhibiting multiple seizures despite initial examinations and diagnostic tests revealing no remarkable findings. She presented with a one-day history of slurred speech and confusion. Her medical history includes hypothyroidism; otherwise, she is in good health.

Collateral history from the husband was obtained. The day before admission, the patient woke up well; however, she experienced episodes of confusion and unresponsiveness. She would make errors such as adding sugar to curry. She also had slurred speech. The following morning, she remained in bed, and when her husband checked on her, she was shaking and had blood in her mouth.

She was initially brought in via the pre-alert stroke pathway, with an initial Glasgow Coma Scale (GCS) score of 10/15 (E4V1M5). The first computed tomography (CT) scan of the head showed no signs of acute haemorrhage, mass lesions, midline shift, or other intracranial abnormalities. On examination, she was moving all four limbs with no lateralisation, she was not following commands, and she was non-communicative. Evidence of tongue biting was also noticed.

The patient was admitted to the emergency department (ED), where she developed a fever and subsequently had two generalised tonic-clonic seizures. The first seizure lasted 10 seconds and self-terminated, while the second seizure lasted 10 minutes. The ED team administered lorazepam, levetiracetam, and phenytoin. Additionally, the patient received ceftriaxone and acyclovir. The initial working diagnosis was meningitis or cerebral venous sinus thrombosis.

**Figure 1:**
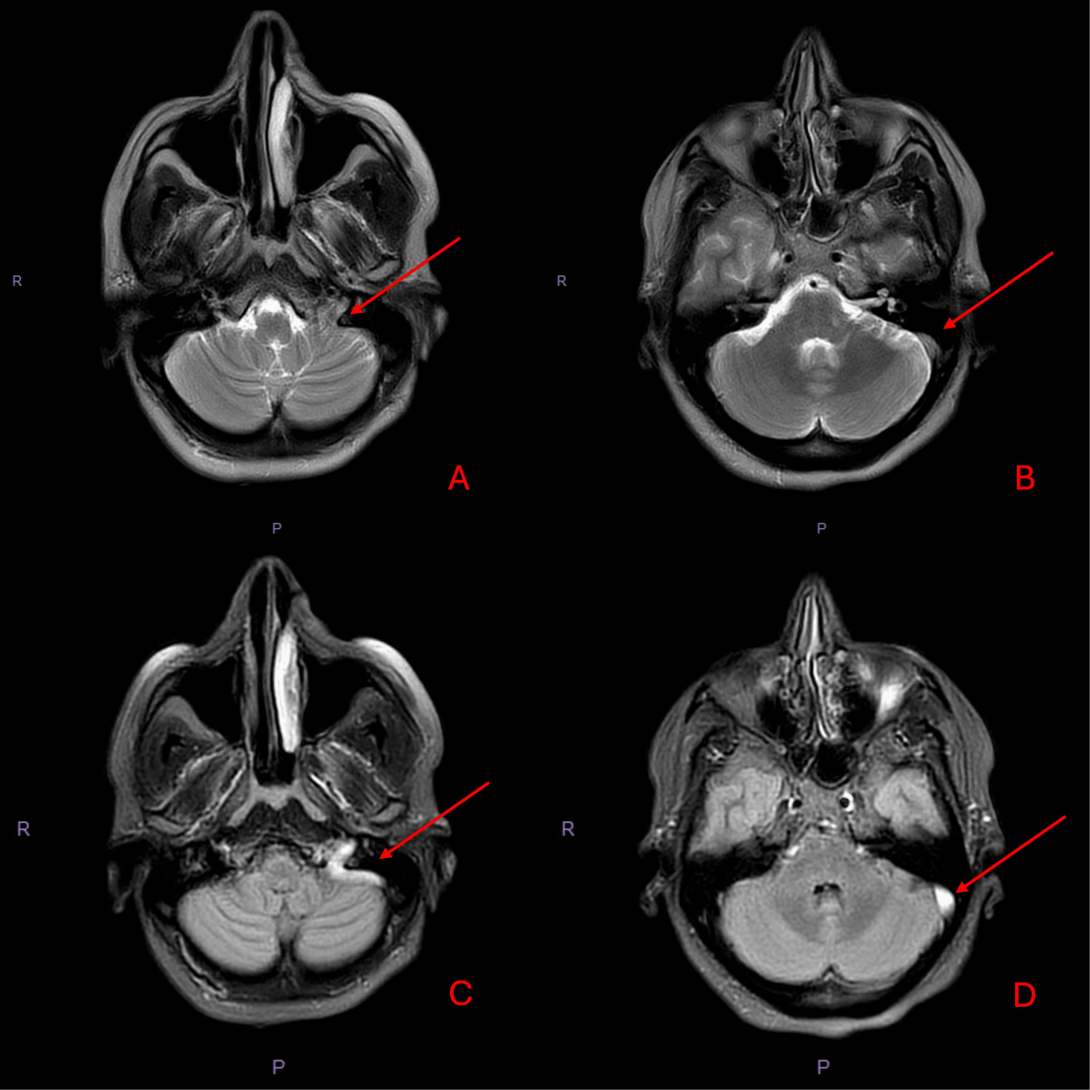
MRI head on admission with equivocal appearances A: T2 MRI with equivocal appearances in the sigmoid sinus (arrow). B: T2 MRI with equivocal appearances in the transverse sinus (arrow). C: FLAIR MRI with equivocal appearances in the sigmoid sinus (arrow). D: FLAIR MRI with equivocal appearances in the transverse sinus (arrow). MRI: magnetic resonance imaging; FLAIR: fluid-attenuated inversion recovery

**Figure 2:**
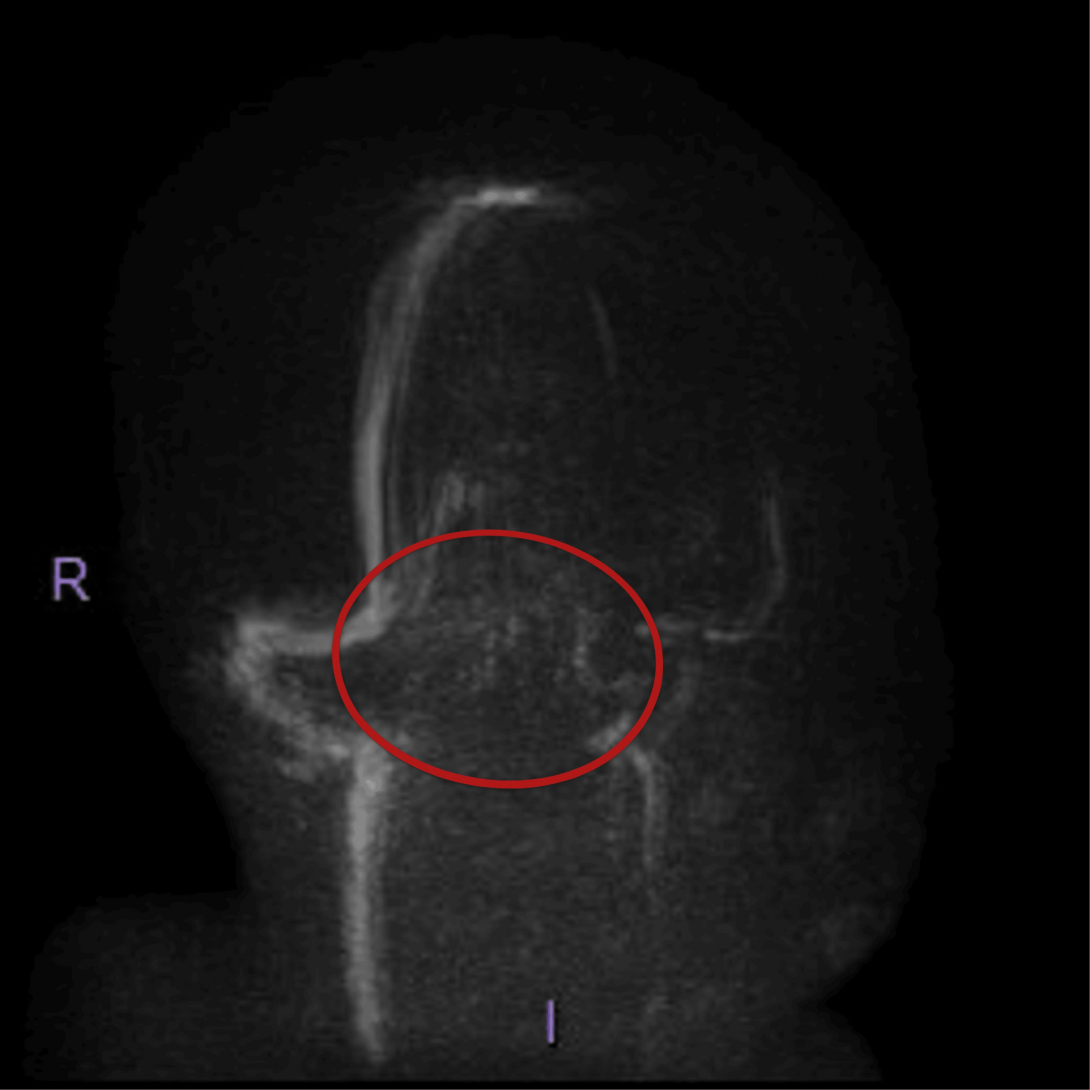
MRI venogram showing signal loss in the left sigmoid and transverse sinuses (red circle)

Due to her low GCS score and her status epilepticus, she was intubated and transferred to the Intensive Therapy unit (ITU). She then underwent magnetic resonance imaging (MRI) of the head and magnetic resonance venography (MRV), neither of which showed evidence of an acute infarct, haemorrhage, or any space-occupying lesions. There were equivocal appearances of the cerebral venous sinuses, specifically the left sigmoid and transverse sinuses, where they displayed intermediate signal on the T2-weighted sequences (Figures 1A-1B) and high signal on the fluid-attenuated inversion recovery (FLAIR) sequence (Figures 1C-1D). There was also signal loss on the MR venogram sequences (Figure 2).

**Figure 3:**
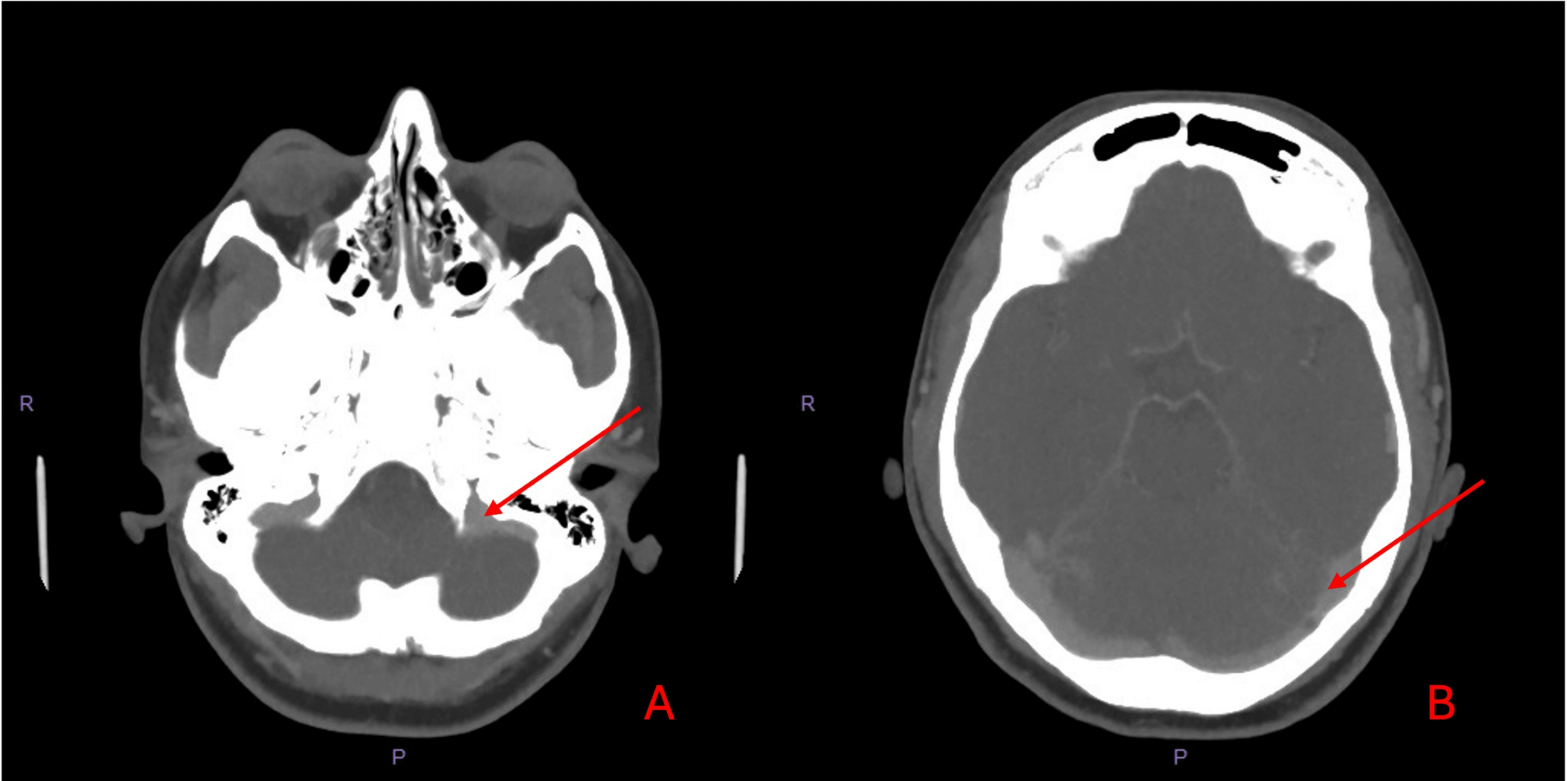
CT venogram ruling out a thrombus in the sigmoid and transverse sinuses A: CT venogram showing no occlusion in the left sigmoid sinus (arrow). B: CT venogram showing no occlusion in the left transverse sinus (arrow).

Thus, a CT venogram (Figure 3) was performed, which effectively excluded the presence of cerebral venous sinus thrombosis.

Day 1 laboratory bloods were unremarkable with a C-reactive protein (CRP) of 0.6 (Table 1). On day 2, there was a rise in CRP to 44, and it later increased to 95 on day 3. The white cell count (WCC) on day 2 was 17.8 x 10^9/L. Her venous blood gas showed lactic acidosis, and there was no growth in her blood cultures.

**Table 1:** Patient’s blood results upon admission including thyroid function tests and thyroid peroxidase antibodies Blood results on the day of admission did not show any significant abnormalities (Table 1). MCH: mean corpuscular haemoglobin; MPV: mean platelet volume; MCV: mean corpuscular volume; RDW: red cell distribution width; NRBC: nucleated red blood cells; MCHC: mean corpuscular haemoglobin concentration; ALP: alkaline phosphatase; ALT: alanine aminotransferase

Parameters	Value	Reference Range
Albumin	39 g/L	35-50 g/L
Calcium	2.33 mmol/L	2.2-2.6 mmol/L
Adjusted calcium	2.35 mmol/L	2.2-2.6 mmol/L
MCH	29 pg	27-32 pg
White cell count	8.1 x 10^9/L	4-11 x 10^9/L
Haemoglobin	139 g/L	120-160 g/L
MPV	9.9 fL	7.8-11 fL
RBC	4.9 x 10^12/L	4-5.2 x 10^12/L
Haematocrit	0.41 L/L	0.36-0.46 L/L
MCV	84 fL	80-100 fL
Platelets	218 x 10^9/L	150-450 x 10^9/L
RDW	17.8%	11.8-14.8%
Neutrophils	5.91 x 10^9/L	2-7 x 10^9/L
Lymphocytes	1.48 x 10^9/L	1-3 x 10^9/L
Eosinophils	0.06 x 10^9/L	0-0.4 x 10^9/L
Monocytes	0.55 x 10^9/L	0.2-1 x 10^9/L
Basophils	0.09 x 10^9/L	0.02-0.1 x 10^9/L
NRBC	<0.5 x 10^9/L	0-0.5 x 10^9/L
MCHC	340 g/L	280-355 g/L
Total protein	71 g/L	60-80 g/L
Calculated globulin	32 g/L	18-36 g/L
Total bilirubin	7 µmol/L	0-20 µmol/L
ALP	59 U/L	30-130 U/L
ALT	8 U/L	0-32 U/L
Magnesium	0.79 mmol/L	0.7-1 mmol/L
C-reactive protein	0.6 mg/L	0-4.9 mg/L
Phosphate	0.95 mmol/L	0.8-1.5 mmol/L
Potassium	3.9 mmol/L	3.5-5.3 mmol/L
Creatinine	67 mmol/L	44-80 mmol/L
Sodium	139 mmol/L	133-146 mmol/L
Urea	3.3 mmol/L	2.5-7.8 mmol/L
Free thyroxine (FT4)	15.9 pmol/L	11-22 pmol/L
Thyroid-stimulating hormone (TSH)	10.8 mIU/L	0.27-4.2 mIU/L
Thyroid peroxidase (TPO) antibodies	323.0 IU/mL	0-33 IU/mL

A cerebrospinal fluid (CSF) analysis obtained via a lumbar puncture showed a total protein concentration of 0.30 g/L, glucose of 4.1 mmol/L, and the absence of identifiable organisms within the CSF (Table 2). The polymerase chain reaction (PCR) analysis of the CSF did not show any evidence of organisms. The CSF WCC was fewer than 5x10^6/L. The viral PCR result came back as negative.

**Table 2:** Patient’s CSF results CSF: cerebrospinal fluid; PCR: polymerase chain reaction

Parameters	Result
Appearance	Clear colourless fluid
Sample volume	1 mL
White blood cells	<5 x10^6/L
Red blood cells	18 x10^6/L
Gram stain	No organisms seen
Organisms	No organisms seen
Culture report	No bacterial growth at 48 hours
CSF PCR	Not detected
*Escherichia coli* K1	Not detected
Haemophilus influenzae	Not detected
Listeria monocytogenes	Not detected
Neisseria meningitidis	Not detected
Streptococcus agalactiae	Not detected
Streptococcus pneumoniae	Not detected
Cytomegalovirus	Not detected
Enterovirus	Not detected
Herpes simplex virus 1	Not detected
Herpes simplex virus 2	Not detected
Human herpesvirus 6	Not detected
Human parechovirus	Not detected
Varicella zoster virus	Not detected
Cryptococcus neoformans/gattii	Not detected

The patient was reviewed by the neurology team, who recommended an electroencephalogram (EEG) and the analysis of both thyroid function tests (TFTs, see Table 1) and N-methyl-D-aspartate (NMDA) receptor antibodies. The patient's TFTs revealed a free thyroxine level of 15.9 pmol/L and a thyroid-stimulating hormone level of 10.8 mIU/L. The NMDA receptor antibodies were negative, and elevated thyroid peroxidase (TPO) antibodies were noted at 323 IU/ml (Table 1). Considering these findings, there was a concern that the patient may potentially be experiencing Hashimoto’s encephalopathy due to the elevated TPO antibodies.

**Figure 4:**
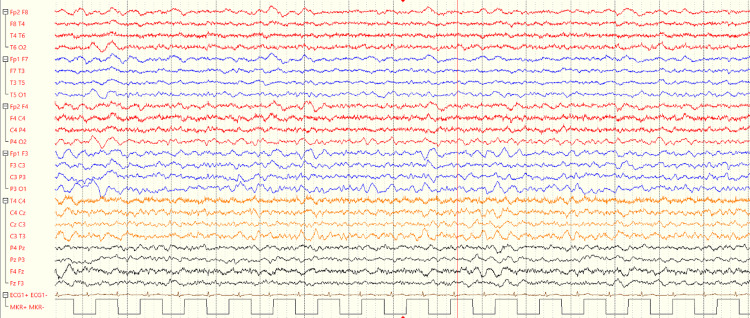
Patient's electroencephalogram (EEG) on day 4 EEG showing the presence of abnormal and fluctuating slowing waves.

She underwent an EEG (Figure 4), which was concluded to be abnormal, showing fluctuating slowing. Overall, this was moderate and diffuse with anterior emphasis, even in the more alert sections. These findings are supportive of an underlying encephalitic process.

**Figure 5:**
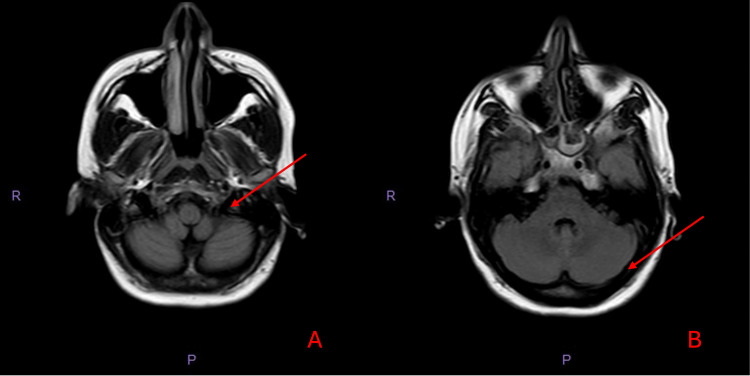
Day 7 MRI head A: Normal appearances of the left sigmoid sinus on FLAIR MRI (arrow). B: Normal appearances of the left transverse sinus on FLAIR MRI (arrow). MRI: magnetic resonance imaging; FLAIR: fluid-attenuated inversion recovery

Following the EEG, the neurology department recommended repeating the MRI of the brain, along with a computed tomography of the chest, abdomen, and pelvis (CTCAP), to rule out malignancy as a possible cause. The CTCAP did not show any signs of malignancy. On the sixth day, she was extubated, and her GCS score on the evening of extubation was 15 out of 15. She was alert, oriented, and following commands. From a circulatory standpoint, she was stable and did not require any supportive measures. On the seventh day, she underwent a repeat MRI of the head (Figure 5), which demonstrated a normal intracranial appearance. The patient was transferred from the ITU to the ward.

The patient's seizures were effectively managed with levetiracetam 1000 mg twice daily, and no additional episodes have been documented since her date of admission. The patient was subsequently discharged home with a plan to continue levetiracetam for seizure prevention.

As part of her follow-up plan after discharge, the patient had a telephone consultation with the neurology team 30 days following her initial presentation. She reported sleep disturbances, feeling generally weak, and anxious. The neurology team wondered if some of these symptoms were related to the side effects of levetiracetam. They scheduled her for a further face-to-face follow-up. She was also reviewed in an outpatient setting by the endocrinology team 41 days after her initial presentation. She reported feeling considerably better but still experienced tiredness and low mood. She was found to have very low vitamin D, which was supplemented. Additionally, her levothyroxine dose was increased. After three months and 20 days, she had a follow-up with the ITU team, where she expressed that she feels like her usual self with no symptoms and no sleep disturbances. Currently, she is only taking an increased dose of levothyroxine. It is noteworthy that the patient failed to attend the scheduled face-to-face follow-up appointment with the neurology department.

## Discussion

Given that the patient exhibited an altered mental status, elevated antithyroid antibodies, and investigations revealed normal CT and MRI brain scans, the patient was deemed to be suffering from Hashimoto’s encephalopathy (Table 3).

**Table 3:** Diagnostic criteria for Hashimoto’s encephalopathy Diagnostic criteria for Hashimoto’s encephalopathy as referenced from Croce et al. [3].

The criteria required for Hashimoto’s encephalopathy to be diagnosed.
1. Encephalopathy with seizures, myoclonus, hallucinations or stroke-like episodes
2. Thyroid disease (subclinical or mild overt)
3. Brain MRI - normal or with nonspecific abnormalities
4. Serum thyroid antibodies present (no specific disease - cut-off value)
5. Absence of other neuronal antibodies in serum or CSF
6. Exclusion of alternative causes of encephalopathy by differential diagnosis

Hashimoto’s encephalopathy was initially documented in the scientific literature in 1966 by Brain et al. [4]. Existing knowledge regarding this condition is limited, with approximately 150 cases reported in the literature [1]. This condition has been referred to by various names in literature; in certain sources, it is named "steroid-responsive encephalopathy associated with autoimmune thyroiditis (SREAT)" [5], and in others, it's referred to as non-vasculitic autoimmune inflammatory meningoencephalitis (NAIM) [6]. The condition is more frequently observed in females than in males [3]. A range of theories regarding the development of Hashimoto’s encephalopathy have been proposed, including its classification as a type of vasculitis, its link to cerebral hypoperfusion, and its recognition as an autoimmune disease.

It's theorised that the underlying factor contributing to the disease is the presence of antibodies that specifically target neuronal components [7]. The investigation conducted by Oide et al. [8] involving patients diagnosed with Hashimoto’s encephalopathy revealed that the serum of these patients contained anti-neuronal autoantibodies. Once again, this supports the theory that an autoimmune component is involved in its pathogenesis.

Patients diagnosed with Hashimoto’s encephalopathy typically exhibit elevated levels of thyroid antibodies, including TPO and anti-thyroglobulin antibodies. Chong et al. [9] suggested that antibody levels do not correlate with the clinical severity of the disease. Thyroid function is typically normal (euthyroid) or, in some cases, may be diminished; hyperthyroidism is an uncommon occurrence.

A study by Ochi et al. [10] identified the autoimmune antigen alpha-enolase in patients with Hashimoto’s encephalopathy. Enolase is a catalytic enzyme present throughout the body, existing in three forms: alpha, beta, and gamma enolases. Through proteome mapping, they found that patients who responded well to corticosteroid treatment had antibodies that reacted with alpha-enolase antigens [10]. Increased levels of anti-alpha enolase antibodies were also observed in patients with the vasculitic condition Kawasaki disease [11], supporting the hypothesis that Hashimoto’s encephalopathy has an autoimmune element in its occurrence. Another study also demonstrated that elevated enolase levels are associated with a lower GCS score [12].

Another theory suggests the pathogenesis indicates a vasculitic phenomenon, leading to localised brain oedema. Studies conducted on patients exhibiting presentations of Hashimoto’s encephalopathy demonstrated areas of hypoperfusion in their brain single-photon emission computed tomography (SPECT) [13].

Individuals afflicted by Hashimoto’s encephalopathy will encounter a spectrum of symptoms, including seizures, confusion, headaches, and mood disturbances [1]. This variability makes Hashimoto’s encephalopathy a challenging condition to investigate due to its diverse range of symptoms. It is believed that Hashimoto’s encephalopathy can manifest in two separate patterns: one characterised by a gradual onset associated with cognitive decline, and the other consisting of focal neurological deficit symptoms [14]. The two types can also have overlapping symptoms [3]. Differential diagnoses may include “rapidly progressing dementia, paraneoplastic and nonparaneoplastic limbic encephalitis” [15].

As previously noted, the presence of high thyroid antibody titres is a crucial diagnostic indicator in Hashimoto’s Encephalopathy. Katoh et al. [16] in 2007 examined an 85-year-old gentleman with Hashimoto’s encephalopathy; his improvement correlated with a reduction in anti-thyroglobulin levels, thereby underscoring the significance of utilising thyroid antibody titres as a diagnostic tool.

Imaging modalities, such as SPECT, can effectively identify regions of hypoperfusion [17]. Brain MRI may reveal areas of oedema [18,19]. CSF can demonstrate elevated protein levels [1,17]. EEG in patients with Hashimoto’s encephalopathy typically presents patterns of “diffuse slow” progression [17].

The utilisation of corticosteroids is the primary therapeutic strategy in managing Hashimoto’s encephalopathy, as evidenced by the symptomatic improvement documented in a case involving a 48-year-old patient, as reported by Chang et al. [20]. They described a case of Hashimoto’s encephalopathy diagnosed in a 48-year-old female patient lacking focal neurological deficits, who only exhibited “cognitive decline” and showed improvement following high-dose corticosteroid therapy [20]. Instances where immunoglobulin therapy has facilitated recovery from Hashimoto’s encephalopathy have been documented [21]. Plasmapheresis has also been considered as an alternative option if steroids fail to induce remission from Hashimoto’s encephalopathy [22].

Similar to our case, there have also been reported cases of spontaneous recovery from Hashimoto’s encephalopathy [16]. Improvement without corticosteroids is rare and has only been reported in a few cases. A systematic review of the literature by de Holanda et al. analysed 130 patients across 52 articles. They identified that in 10 cases, there was remission without treatment [23]. Tang et al. [24] analysed 13 patients, five of whom did not receive corticosteroid treatment. The majority of patients undergoing corticosteroid therapy demonstrated improvement. Among the five patients who did not receive corticosteroid therapy, two were treated with antiepileptic medications and did not experience subsequent seizures.

Our patient did not receive any corticosteroids; she was managed with the antiepileptic medication levetiracetam for her seizures. Following extubation, her predominant neurological symptoms, namely seizures, confusion, and behavioural changes, resolved. She was discharged on levetiracetam, which has since been discontinued, and she has not experienced any further seizures. Her dose of levothyroxine was increased. She was still experiencing sleep disturbances, which resolved approximately four months later.

## Conclusions

The concept of Hashimoto’s encephalopathy remains not fully understood. Despite exhibiting characteristic features of Hashimoto’s encephalopathy, including seizures, confusion, behavioural changes, and elevated anti-TPO antibodies, our patient achieved complete clinical recovery without the use of immunosuppressive therapy. While corticosteroids are regarded as the primary treatment modality, as indicated by the majority of existing literature, clinicians should be aware of the potential for spontaneous remission. Consequently, although the clinical manifestations of Hashimoto’s encephalopathy may vary, we implore that it’s imperative to consider Hashimoto’s encephalopathy in the differential diagnosis when focal neurological deficits, psychiatric symptoms, and seizures occur in the absence of an apparent organic cause.
